# E‐cigarettes in Nigeria: A scoping review of evidence

**DOI:** 10.1002/hsr2.2074

**Published:** 2024-04-24

**Authors:** Oluwatobi E. Adegbile, Oluwatomilayo D. Adeniji, Jimoh Amzat, Kehinde K. Kanmodi

**Affiliations:** ^1^ Department of Biostatistics and Epidemiology East Tennessee State University Johnson City Tennessee USA; ^2^ Campaign for Head and Neck Cancer Education (CHANCE) Programme Cephas Health Research Initiative Inc Ibadan Nigeria; ^3^ Department of Community and Behavioral Health East Tennessee State University Johnson City Tennessee USA; ^4^ Department of Sociology Usmanu Danfodiyo University Sokoto Nigeria; ^5^ Department of Sociology University of Johannesburg Johannesburg South Africa; ^6^ Faculty of Dentistry University of Puthisastra Phnom Penh Cambodia; ^7^ School of Dentistry University of Rwanda Kigali Rwanda; ^8^ School of Health and Life Sciences Teesside University Middlesbrough UK

**Keywords:** e‐cigarette, electronic nicotine delivery system, Nigeria, scoping review, vaping

## Abstract

**Background and Aims:**

E‐cigarettes will continue to be a public health issue in Nigeria. To curb the growing menace of the e‐cigarette use in Nigeria through evidence‐based approach, it is crucial to first map the empirical research landscape of e‐cigarettes in Nigeria. No known study has mapped the existing empirical evidence and gaps concerning e‐cigarettes in Nigeria; hence, this scoping review was conducted.

**Methods:**

This scoping review adopted the research design by Arksey and O'Malley. Four databases (PubMed, SCOPUS, CINAHL Complete, and APA PsycINFO) were searched to retrieve literature on e‐cigarettes in Nigeria. With the aid of Rayyan web application, all retrieved literature were deduplicated and screened based on the review's eligibility criteria. Only those peer‐reviewed journal papers meeting the inclusion criteria were included in the review. Relevant data from the included papers were charted, collated, and summarized.

**Results:**

A total of six papers were included in this review. The reviewed papers reported a lifetime prevalence of e‐cigarette use (or vaping) ranging from 5.8% to 19.8%, with a current e‐cigarette use prevalence of 11.8%, among different population groups in Nigeria. The major determinants of e‐cigarette use, as reported in these articles, include being a youth, having a health condition, severe anxiety, tobacco use, peer influence, and current alcohol use. Dry mouth and oral lesions (gingival inflammation and oral ulcers) were also identified to be the medical conditions associated with e‐cigarette use in Nigeria. Lastly, one of the included papers identified a lack of clear regulation on e‐cigarettes in Nigeria.

**Conclusion:**

There is an urgent need for more scientific investigations on the sociodemographic, economic, health, and regulatory landscape of e‐cigarettes in Nigeria, as robust empirical evidence is needed for the effective planning, implementation, and evaluation of evidence‐based policies and interventions on e‐cigarettes control and regulation in Nigeria.

## 1 INTRODUCTION

Electronic cigarettes (e‐cigarettes), also referred to as electronic nicotine delivery systems (ENDS), are electronic or battery‐operated devices that vaporize a liquid (typically consisting of propylene glycol and glycerol, with or without nicotine and flavors) into an inhalable aerosol.^1^ The liquid is contained in disposable or refillable cartridges or reservoirs.^1^ Vaping, a process of using an e‐cigarette produces feelings comparable to smoking a combustible cigarette both in taste and similar pleasures that smoking generates.^1^ Newer e‐cigarette devices have evolved in shape and variety, often looking like pens and other contemporary appearances compared to older versions that resembled cigarettes. Hence, e‐cigarettes are heterogeneous in terms of devices, products, e‐liquids, and product components.^1^ Although the potential impact on e‐cigarette users is still uncertain,^2^ existing evidence had identified numerous chemical components present in e‐liquids, cartridges, and e‐cigarette aerosols including aldehydes, nicotine, and metals.^3^, ^4^, ^5^, ^6^, ^7^

The introduction of e‐cigarettes for retail sale was initially to help combustible cigarette smokers quit smoking^8^, ^9^; however, there has been rapid uptake of e‐cigarette use by noncombustible smokers, teenagers, and young adults.^10^, ^11^, ^12^ Introduced around the early 2010s, e‐cigarettes gained popularity as a perceived safer alternative to traditional tobacco products. However, their rapid adoption, particularly among youth demographics, has raised concerns about potential health risks and regulatory challenges.^13^, ^14^ Monitoring e‐cigarette trends helps to assess patterns of use and misuse and facilitate proactive regulatory policies.^15^ Among young adults (aged 19–30 years) in the United States, e‐cigarette use prevalence in 2021 was 21.8% (vaping nicotine) and 18.7% (vaping marijuana) reflecting over a 100% increase when compared to 2017 estimates.^12^ In a 2021 survey among adults in Canada, e‐cigarette ever‐use prevalence was 48% (aged 20–24 years), 29% (aged 15–19 years), and 13% (aged ≥25 years).^16^ Among all adults in Great Britain, e‐cigarette prevalence has remained constant since 2015; however, 2017–2018 prevalence estimates account for 5.4% to 6.2% for all adults.^17^ In Southeast Asia, the prevalence of current e‐cigarette users varies from 3.3% (Thailand) to 11.8% (Indonesia).^18^

The addictive effect that occurs from e‐cigarettes underscores the potential for e‐cigarette use to exert a more profound impact on the developing brain, heightening susceptibility to the use of other substances.^19^, ^20^, ^21^ This is important as critical brain regions do not reach full maturity until individuals are in their early or mid‐20s.^19^ Similarly, an essential component of e‐cigarette use is recognizing the appropriate time to quit. The prefrontal cortex, which governs judgment, decision‐making, behavior, and emotion control, continues to undergo development throughout adolescence. As a result, teenagers often face difficulties in determining when to quit e‐cigarette experimentation, as their ability to accurately assess risks is not fully matured.^20^, ^21^, ^22^ Ultimately, this elevates their risk towards re‐using or continuous e‐cigarette use, use of other substances, and a stronger tender to substance use disorders.^20^, ^23^, ^24^, ^25^ Diverse perspectives exist regarding the potential harm reduction value of e‐cigarettes for adults, particularly for combustible cigarette smokers.^8^, ^9^ Nevertheless, there is widespread agreement on the necessity to safeguard young people from the potential risks associated with this emerging technology.^26^

Recent evidence has highlighted potential environmental concerns associated with e‐cigarette use,^27^ including mixed evidence regarding potential health risks. Some research findings are indecisive on the future health impacts of e‐cigarette use^28^, ^29^, ^30^ while other findings have advanced potential for cardiovascular diseases^31^, ^32^, ^33^ or respiratory diseases occurrence.^34^, ^35^, ^36^ In the interim, significant fatality was recorded from “e‐cigarette or vaping product use associated lung injury” (EVALI) in the United States in 2019 when e‐cigarette e‐liquids were used in combination with marijuana oil leading to the deaths of over 65 people who vaped this product including 2800 hospitalizations.^37^ Furthermore, conflicting findings exist among published evidence between 2018 and 2020 regarding e‐cigarette effectiveness as a tool to quit smoking. Some findings suggest that e‐cigarettes were found to be effective in helping smokers quit,^38^, ^39^, ^40^ other studies suggest that e‐cigarette use prevented smokers from quitting^40^, ^41^, ^42^ while other studies presented inconclusive evidence.^43^, ^44^, ^45^

Despite varying evidence to support or prevent e‐cigarette use, it continues to appeal to several consumers—mostly teenagers and young adults. Factors such as innovative brands, tailored advertisements to youths, especially on social media, and the varieties of e‐cigarette flavors and products have accounted for the wide uptake of e‐cigarettes.^46^, ^47^, ^48^ Limited advisory on age and health risks associated with e‐cigarette use as seen in online advertisements alongside potential harm associated with nicotine dependence escalates the dire need for a robust governmental policy in providing regulatory guidance to marketers and consumers of e‐cigarette.^23^, ^24^, ^25^, ^48^, ^49^ E‐cigarette sales have deeply penetrated developed economies like the United States.^50^ With increasing regulation of the e‐cigarette market in developed countries, marketers are looking to expand their business to developing economies where borders are porous and regulations less stringent.^51^, ^52^, ^53^ Nigeria, a low‐middle‐income country with a vast youthful population is a prime target for this expanding e‐cigarette market.

The use of e‐cigarettes in Nigeria has been fueled by numerous factors including aggressive marketing strategies, accessibility, and the allure of trendy vaping culture. Amidst limited tobacco control policies and enforcement mechanisms, the vaping industry expanded rapidly, with a plethora of brands and flavors flooding the market. Despite the absence of comprehensive national data, anecdotal evidence suggests a growing prevalence of e‐cigarette use among Nigerian youths, with concerns about nicotine addiction and long‐term health implications.^13^, ^14^ Additionally, the lack of regulation has facilitated the proliferation of substandard products and counterfeit imitations, aggravating health risks. To strengthen governmental commitment towards robust policy formulation and funding of evidence‐based prevention programs, there is a need to contextualize the burden of e‐cigarette use in Nigeria. Different studies have been conducted to investigate multidisciplinary and contemporary issues concerning e‐cigarette practices in Nigeria.^54^, ^55^, ^56^, ^57^, ^58^, ^59^, ^60^, ^61^, ^62^ However, no known study in Nigeria has charted, collated, and summarized all research evidence, as well as identified inherent gaps found in existing literature. Therefore, this study aims to conduct a scoping review to address these concerns. The findings from this review will provide relevant insights into the current epidemiology and impact of e‐cigarette use in Nigeria especially among at‐risk populations (adolescents and young adults) as well as provide directions for future risk and market surveillance, research on health impacts, policy responses, evidence‐based prevention programs, and stakeholders engaged in clinical and/or public health practice.

## 2 METHODS

### 2.1 Review design

The design of this review was based on the stepwise procedure, developed by Arksey and O'Malley, for conducting scoping reviews.^63^ Also, we followed the Preferred Reporting Items for Systematic Reviews and Meta‐analyses extension for Scoping Reviews guidelines (PRISMA‐ScR) in reporting this review.^64^

### 2.2 Research question identification

This study seeks to answer this principal research question: What scientific evidence exists on e‐cigarette or vaping in Nigeria? However, to obtain answers on some pertinent areas, the principal question was divided into six subquestions:

Subquestion 1: What scientific evidence exists on the awareness and knowledge of e‐cigarette or vaping in Nigeria?

Subquestion 2: What scientific evidence exists on the prevalence of e‐cigarette use in Nigeria?

Subquestion 3: What scientific evidence exists on the determinants of e‐cigarette use in Nigeria?

Subquestion 4: What scientific evidence exists on the health implications of e‐cigarette use in Nigeria?

Subquestion 5: What scientific evidence exists on the public health interventions (including policy) on e‐cigarette or vaping in Nigeria?

Subquestion 6: What other scientific evidence exists on e‐cigarette or vaping in Nigeria?

### 2.3 Literature search

On January 27, 2023, a systematic search was used to retrieve relevant literature from the PubMed, SCOPUS, CINAHL Complete, and APA PsycInfo databases. For the achievement of robust literature selection, the search for studies in the listed electronic databases was developed from the appropriate research terms and best fit to answer the research questions developed for this scoping review. This search was conducted using relevant terms and synonyms (“vape” OR “vaping” OR “e‐cigarette” OR “electronic nicotine delivery”) AND (“Nigeria”) obtained from the Medical Subject Heading (MeSH) dictionary and Thesaurus, and with the aid of Boolean operators (“AND” and “OR”), to retrieve all relevant literature published from inception till December 2022. Supporting Information S1: Tables S1–S4 show the search strategy used for the search from each of the selected databases. Also, we did a manual search of the reference lists of the literature that were included in this review to further retrieve any other eligible literature that were not identified through a database search.

Only those publications that met the following criteria were included in the review:

- papers published in refereed journals;
- journal papers of any type (letters, editorials, comments, original research articles, review articles, etc.);
- journal papers reporting empirical evidence concerning e‐cigarettes or vaping in Nigeria; and
- journal papers with accessible full text.

**Figure 1:**
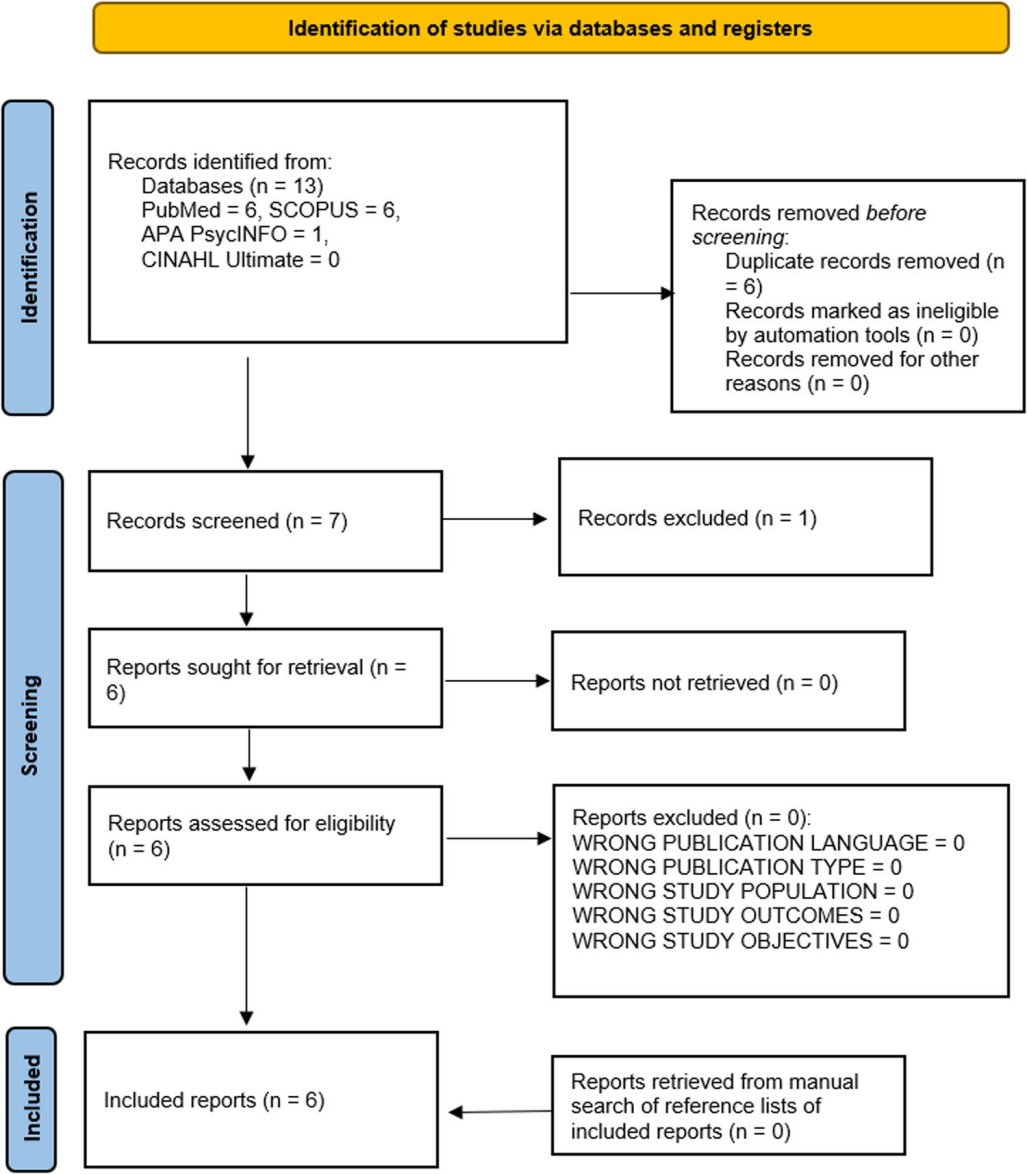
PRISMA flowchart diagram. PRISMA, Preferred Reporting Items for Systematic Reviews and Meta‐analyses.

The citations of the literature obtained from the database search were exported into the Rayyan software for deduplication. After deduplication, the residual literature was screened for inclusion in the review (Figure 1). The screening process was two‐staged and done by two reviewers (one medical doctor [O. E. A.] and one dental surgeon [K. K. K.], both with specializations in the field of public health). The review's inclusion criteria guided the screening process, with the first stage involving the title and abstract screening while the second stage involved full‐text screening. Only those articles that met the inclusion criteria were included in the review. A third reviewer (O. D. A.) was involved in situations where there were conflicts in the decisions of the two reviewers (O. E. A. and K. K. K.). Additionally, there was no ambiguity encountered in interpreting abstracts during the screening process; hence, no author was contacted to provide clarification or additional information on the screened literature.^65^

### 2.4 Quality appraisal

The Mixed Methods Appraisal Tool (MMAT) 2018 version was used to appraise the quality of the included literature,^66^ and two reviewers (O. E. O. and K. K. K.) were involved in the appraisal process. The MMAT uses a set of seven questions per research design to appraise empirical research literature. This research design appraisal tool could assess qualitative study designs (ethnography, phenomenology, grounded theory, etc.), quantitative randomized controlled trial designs, quantitative nonrandomized study designs (nonrandomized controlled trial, cohort study, case‐control study, and cross‐sectional analytical study), quantitative descriptive study designs (case series, case report, etc.), and mixed methods study design (convergent study, sequential exploratory study, and sequential explanatory study).^66^ For each of the questions in the MMAT, there are three possible answers: “Yes,” “No,” and “I can't tell.”^66^ In the appraisal process, a reviewer's response of “Yes” was awarded 1 point, a response of “No” was awarded zero point, and a response of “I can't tell” was awarded zero point. Since empirical research literature can only be graded with seven questions, a 7‐point scale was therefore used to grade the appraisal outcome. Appraised literature with a grade of 4/7 points or above were considered to be of “above average” quality while those with a grade of 3/7 points or below were considered to be of “below average” quality. However, all included literature were reviewed in this scoping review regardless of their quality appraisal outcomes (Supporting Information S1: Tables S5–S7).

### 2.5 Data charting

Following rigorous deliberations and standardization, three reviewers (O. E. A., O. D. A., and K. K. K.) collaborated to extract data from the included literature. From the included literature, the following data were extracted: citation details (names of authors and the year of publication), journal paper type, study design, study population size and characteristics (gender distribution, age distribution, and other sociodemographic features), study location, study findings, and conclusions.

### 2.6 Collation, summarization, and presentation of the charted data

The charted data are collated and summarized using a narrative synthesis approach. The summarized data are thereafter presented in texts and a table (Table 1).

**Table 1:** Summary of the included literature.

No.	Reference	Publication type	Study design	Study location (in Nigeria)	Objectives	Study instruments	Population characteristics	Conclusion
1	Omaiye et al.^67^	Journal paper (original research article)	Quantitative cross‐sectional study	Abuja City and Lagos State	To compare nicotine concentrations in one brand (Ritchy Group Limited) of refill fluids that were purchased in four countries and labeled in Nigeria 0 mg of nicotine/mL.	High‐performance liquid chromatography machine; ICONIT software (full meaning of ICONIT not stated in the paper)	Study population: Electronic cigarette refill fluids purchased in Nigeria, the United States, England, and China Sample size: 125 (Nigeria—29 refill fluids in seven flavors; United States—61 refill fluids in 50 flavors; England—eight refill fluids in eight flavors; China—27 refill fluids in 25 flavors).	There is a need for better monitoring and control of nicotine‐containing products and the sales outlets of these products in Nigeria.
2	Alade et al.^68^	Journal paper (original research article)	Quantitative cross‐sectional study design	36 States of Nigeria and the Federal Capital Territory.	To assess the oral lesions associated with the use of e‐cigarettes, tobacco smoking, and COVID‐19 among adolescents and young people in Nigeria.	Electronic questionnaire	Study population: Adolescents and young people Sample size: 2870 Age range: 11‐ to 23‐year‐old Gender: male—1449 (50.5%); female—1421 (49.5%) Race/Ethnicity: not stated. Socioeconomic status: not stated	The results obtained from the study were significant. The findings May be helpful for clinical screening for COVID‐19 and tobacco use among young people (including adolescents) in Nigeria.
3	Folayan et al.^61^	Journal paper (original research article)	Quantitative cross‐sectional study design	36 States of Nigeria and the Federal Capital Territory.	To determine the proportion of adolescents and young persons (AYP) who use e‐cigarettes and smoke tobacco. To identify differences in factors associated with the use of e‐cigarettes and tobacco smoking.	Electronic questionnaire	Study population: Adolescents and young persons aged 11–23 years. Sample size: 2206 Mean age: not stated. Gender: male—1449 (50.5%); female—1421 (49.5%) Ethnicity: not stated. Educational status: secondary school or less—1576 (54.9%); university or higher—1294 (45.1%)	The prevalence of e‐cigarette and tobacco use among adolescents and young persons in Nigeria is high. There is an urgent need for public health interventions targeting the common risk factors for the use of e‐cigarettes and tobacco.
4	Erinoso et al.^54^	Journal paper (original research article)	Quantitative cross‐sectional study design	Lagos State	To assess the prevalence and factors associated with electronic cigarette use, as well as the relationship between their use and anxiety among adolescents and young adults in Lagos, Nigeria.	Electronic questionnaire	Study population: Individuals aged 15–35 years. Sample size: 949 Mean age: 23.36 (SD = 3.97) years. Gender: male—421 (44.4%); female—528 (55.6%) Ethnicity: not Stated Educational status: below secondary school—2 (0.2%); secondary school—112 (11.8%); college and above—832 (88.0%)	The findings in this study suggest a higher likelihood of e‐cigarette use among poly‐tobacco or substance users, individuals with friends who use e‐cigarettes, and alcohol consumers. Health providers and policymakers in Nigeria might consider preventive measures targeting young adults with the identified risk factors, as well as close monitoring of the trends in the use of e‐cigarette in the coming years.
5	Erinoso et al.^68^	Journal paper (letter to editor)	Quantitative cross‐sectional study design	Online (geographical location in Nigeria not specified)	To advance research about the online retail environment for ENDS in relatively understudied markets by surveying online retailers that sell ENDS in Nigeria and assessing product types and e‐liquids.	Not clearly defined.	Study population: online retail stores with a contact address in Nigeria having one or more electronic nicotine delivery systems (ENDS) or other product reviews. Sample size: 20 stores	There is a need for policymakers in Nigeria to consider precautionary regulations for ENDS products to curb the indiscriminate activity of ENDS products industry and protect health
6	Osibogun et al.^62^	Journal paper (original research article)	Qualitative (exploratory) study design	Lagos State	To explore the knowledge and risk perception of e‐cigarettes and hookah among young people in Lagos, Nigeria	Question guide (format not stated); audio recorder	Study population: Young people in Lagos who had used e‐cigarettes or hookah at least once in the past 12 months Sample size: 20 Age: range—15–24 years; mean—20.4 years Gender: male—15 (75%); female—5 (25%) Ethnicity: not stated Socioeconomic status: not stated	Young people using e‐cigarettes and hookah continued to use these products despite their awareness of some associated harmful health effects. Stress relief and social reasons were the primary purpose behind the use of these products. The creation of awareness of the hazards associated with the consumption of e‐cigarettes and hookah may help to reduce the acceptability of these products among Nigerian youths.

Abbreviation: SD, standard deviation.

## 3 RESULTS

### 3.1 Literature search, deduplication, and screening outcomes

Thirteen literature were retrieved from the literature search [PubMed = 6; SCOPUS = 6; APA PsycInfo = 1; CINAHL Ultimate = 0]. Out of these literature, six were duplicates and were deleted. The titles and abstracts of the remaining seven publications were screened, and one nonrelevant publication was excluded. The full texts of the remaining six literature were screened, and all were relevant and included in the scoping review. In addition, the reference lists of the included literature were manually searched but no eligible literature was found (Figure 1).

### 3.2 Quality appraisal outcomes

All the included literature were appraised, and they had a grade of 6/7 points and above; hence, they were all considered to be of above‐average quality (Supporting Information S1: Tables S5–S7).

### 3.3 Publication trend by year

All the included literature were journal papers published within the last 6 years, of which one was published in 2017,^67^ one in 2020,^62^ one in 2021,^54^ and three in 2022^59^, ^61^, ^68^ (Table 1).

### 3.4 Publication type

All the included papers, of which five^54^, ^59^, ^61^, ^62^, ^67^ were original research articles while one^68^ was a letter to the editor (Table 1).

### 3.5 Study design

None of the included papers adopted an experimental or quasiexperimental research design. However, five papers^54^, ^59^, ^61^, ^62^, ^67^ adopted a quantitative cross‐sectional study design while one^62^ adopted a qualitative study design (Table 1).

### 3.6 Study location

All the included papers reported the specific geographical location in Nigeria where their study data were collected, except for the study by Erinoso et al.^68^ which referred to no specific Nigerian location. Among the remaining five papers, two^54^, ^62^ collected data from Lagos, one^67^ collected data from Lagos and Abuja, and two^59^, ^61^ collected data from all 36 states in Nigeria including the Federal Capital Territory (Table 1).

### 3.7 Study instrument

All the included papers specifically reported their study instrument, except for the one by Erinoso et al.^68^ which was not clearly defined. Of the remaining five papers, one adopted the use of a high‐performance liquid chromatography machine for data collection, one adopted the use of ICONIT (full meaning not stated in the reviewed article) software, three adopted the use of a questionnaire, one adopted the use of a question guide, and one adopted the use of an audio recorder (Table 1).

### 3.8 Study population

The included papers investigated diverse populations. Only one paper investigated e‐cigarette refill fluids, one investigated retail stores, and four investigated a combined population of adolescents and young adults. In total, 3839 persons aged 11–35 years, 29 e‐cigarette refill fluids, and 20 retail stores in Nigeria were investigated (Table 1).

### 3.9 Awareness and knowledge of e‐cigarettes in Nigeria

Two papers provided evidence of the awareness and knowledge of e‐cigarettes in Nigeria. One of the papers, by Erinoso et al.^54^ utilized a quantitative approach while the other paper, by Osibogun et al.,^62^ utilized a qualitative approach with both papers investigating a total of 969 adolescents and young adults. Only the study by Erinoso et al.^54^ reported awareness of e‐cigarettes as 59.7%.

### 3.10 Prevalence of e‐cigarette use in Nigeria

Three papers provided evidence of the prevalence of e‐cigarette use in Nigeria, and all three studies surveyed a combined population of adolescents and young adults.^54^, ^59^, ^61^ All three papers reported different lifetime prevalence rates which ranged from 5.8% to 19.8%.

Also, it is noteworthy that only one paper, by Erinoso et al.,^54^ reported the prevalence of current use (i.e., use within the past 30 days) of e‐cigarettes in Nigeria. In their study, 11.8% (6/51) of participants with a lifetime history of e‐cigarettes were found to be current e‐cigarette users.

### 3.11 Determinants of e‐cigarette use in Nigeria

Three papers^54^, ^61^, ^62^ provided evidence about the determinants of e‐cigarette use or vaping in Nigeria. In those papers, the following determinants were associated with e‐cigarette use: male gender (adjusted odd ratio [AOR]: 1.22),^61^ older youths (AOR: 1.05–3.35),^54^, ^61^ presence of a health condition including human immunodeficiency virus (HIV) (AOR: 1.51–1.90),^61^ high anxiety (AOR: 0.86–1.87),^54^, ^61^ COVID‐19 infection (AOR: 3.60),^61^ being vulnerable (AOR: 2.00),^61^ high school education (AOR: 2.08),^61^ tobacco use (AOR: 2.18–3.55),^54^, ^61^ peer influence (AOR: 2.72–4.43),^54^, ^61^ familiar factors (father, mother, siblings—AOR: 1.52–2.33),^61^ current alcohol intake (AOR: 5.49),^54^ and as a harm reduction tool.^62^

### 3.12 Health implications of e‐cigarette use in Nigeria

Only one paper^59^ provided evidence of the health implications of e‐cigarette use in Nigeria. This paper identified oral lesions (gingival inflammation AOR: 1.51,^59^ oral ulcers AOR: 1.89),^59^ and dry mouth (AOR: 1.96)^59^ as some of the health implications of e‐cigarette use in Nigeria.

### 3.13 Public health interventions (including policy) on e‐cigarettes and vaping in Nigeria

None of the included papers provided evidence of public health interventions (including policy) on e‐cigarette use and vaping in Nigeria.

### 3.14 Other findings on e‐cigarettes and vaping in Nigeria

Two papers^67^, ^68^ provided other findings on e‐cigarettes and vaping in Nigeria. One paper, by Omaiye et al.,^67^ reported on counterfeit electronic cigarette refill fluids purchased from online stores in Abuja, Nigeria labeled 0 mg/mL of nicotine but contained nicotine in quantities ranging from 3.7 to 20.4 mg/mL following analysis of the e‐cigarette refill fluid. The second paper by Erinoso et al.^68^ reported on the online retail environment for electronic nicotine delivery systems (ENDS) in Nigeria. This paper stated that seven out of 20 (35%) online retail stores surveyed exclusively sold ENDS products and e‐liquids. A majority (75%) of the online retail stores surveyed in this paper provided access to PODS sales while over 65% of the e‐liquid products sold provided no label information regarding the nicotine concentration of the e‐liquids or age restrictions/advisory regarding its use.

## 4 DISCUSSION

This scoping review is believed to be the first study to broadly collate all existing information available on e‐cigarette in Nigeria, and the identified findings are noteworthy and insightful for public health planning, research, practice, and policy and/or regulation formulation. Based on this review's findings, it can be noted that most of the population groups that were surveyed in Nigeria were adolescents and young adults (ages 11–35 years). Similarly, the identified lifetime prevalence of e‐cigarette use ranged from 5.8% to 19.8% while the identified prevalence of current use of e‐cigarettes was 11.8%. The e‐cigarette use prevalence range reported in this review is lower than that reported in a South African study, where a prevalence of 37% e‐cigarette use was reported among a comparable population group.^69^ Cumulatively, this shows that e‐cigarette use is becoming a popular practice among adolescents and young adults in Africa.

According to the 2012 National Bureau of Statistics in Nigeria, the population of individuals aged 15–35 years in Nigeria was estimated to be about 64 million people; this represents about 36.8% of the productive workforce population of Nigeria.^70^ As earlier described (see the introduction section of the full text), early‐onset e‐cigarette use could facilitate the use of other illicit or psychoactive substances.^23^, ^24^ Upon leaving high school and gaining more independence, whether in college or as employed adults, individuals might be exposed to e‐cigarette use without the protective framework provided by family and school resulting in profound consequences including use/high‐risk use of other substances (combustible cigarettes, heroin, smoking marijuana, and prescription stimulants used nonmedically). Consequently, this may heighten the frequency of drug use, misuse, and addiction, leading to harmful health outcomes, particularly chronic diseases (HIV and hepatitis B and C).^28^, ^71^ A significant proportion of older adults who meet the criteria for a substance use disorder began using substances during their teenage and young adult years.^22^ Furthermore, substance misuse is associated with risky sexual behaviors, exposure to violence, violent crimes (homicides and rape), and increased risks of mental health issues and suicide.^72^ These deleterious outcomes have profound negative impacts on college education enrollment, engagement, retention, and success of Nigerian students who are predominantly adolescents and young adults^55^ thereby highlighting the urgency of addressing vaping practices among this at‐risk population group.

Reports in other climes have shown that e‐cigarette use among tertiary institution students could lead to future traits of impulsivity, risky behaviors, and increased use of other illicit drugs like opioids, cocaine, and other stimulants or hallucinogens which may result in anxiety, low self‐esteem, and defiant behaviors including gambling.^23^ These outcomes may impact significantly the psychological and socioeconomic contributions of youths to national growth and development especially in a country like Nigeria. Furthermore, mental health outcomes including depression and suicidal ideation have been associated with adolescents' e‐cigarette use.^73^ Overall, the above‐identified risks lend insights into the potential dangers associated with adolescent/youth e‐cigarette use on student retention, drop‐out, and future career development within higher education and/or workplace settings in Nigeria.

Clearly, from the available empirical research data obtained in this scoping review, there was limited information to highlight the prevalence trends of e‐cigarette use in Nigeria. However, this paucity of data is not limited to Nigeria alone, such was also observed in other sub‐Saharan African countries.^74^ Hence, it would be highly beneficial if research efforts were geared towards continuous data collection on e‐cigarette use in Nigeria and other sub‐Saharan African countries as well. By so doing, this would help inform policy direction and predict evidence‐based interventions to enhance e‐cigarette cessation, addiction prevention, and harm reduction, especially within the at‐risk populations, more especially the adolescent and young adult population group. Additionally, the impact of the COVID‐19 pandemic continues to heighten e‐cigarette use^37^ with a proportional increase in adolescent and young adults' engagement in risky behaviors. Hence, it is paramount to continuously collect relevant data to inform policy direction that would limit the risk indices in at‐risk users. Going forward, this can be achieved in Nigeria through the establishment of a national database to monitor future trends in e‐cigarette use and how it impacts the Nigerian population, especially its productive workforce.

Notably, this scoping review also identified the determinants of e‐cigarette use among the Nigerian population, and being an older youth, having a health condition, severe anxiety, previous or current tobacco use, peer influence, and current alcohol use were the major determinants identified.^54^, ^61^, ^62^ In the course of comparing our findings with those that have been reported in other African countries, through an extensive literature search, we found that no other known study was found to have exclusively reported the determinants of e‐cigarette use in Africa.^69^, ^75^ This observation highlights a major research gap that should be a focus for future research considerations in Nigeria and Africa at large. Pertinently, several other determinants may predict e‐cigarette use in Nigeria that are yet to be identified through empirical research. Hence, policy experts, research institutions, and government agencies need to commit extensive resources to fully investigate the roles that family (family tradition, religious beliefs, cultural values), socioeconomic (education, employment, income), behavioral, and biological factors play in determining the initiation, continued use, and quit tendencies for e‐cigarette in Nigeria.

Additionally, this scoping review identified the health implications of e‐cigarette use among the Nigerian population. Two of the included papers reported how vaping could lead to smoking.^61^, ^62^ While this finding demands further exploration, it portrays grave danger to the success of existing tobacco legislation in Nigeria. Consequently, there is a need for researchers, public health experts, and policy analysts to further investigate these trends and provide timely insights critical to enhancing policies aimed at smoking cessation in Nigeria. Furthermore, this review also identified other health implications of e‐cigarette use in Nigeria which include oral lesions (gingival inflammation, oral ulcers), lung conditions, and liver disease. However, the implications identified in these studies were not exhaustive. For example, several studies outside Nigeria have identified that e‐cigarette use can cause e‐cigarette, or vaping use–associated lung injury (EVALI).^37^, ^76^ To broadly understand the diverse health implications of e‐cigarette use in Nigeria, its specific causes, and future complications of these conditions, clinicians, public health experts, researchers, policy analysts, and government agencies need to develop and formulate strategic coalitions that will bolster effective and well‐coordinated approaches to collect relevant data and design evidence‐based programs to reduce e‐cigarette use and its continued attendant effect on the human health.

It is noteworthy that this review also identified some empirical evidence on issues concerning e‐cigarette regulation in Nigeria.^68^ Findings suggest that there are no effective laws or policies tailored to regulating e‐cigarettes in Nigeria. In the reviewed study,^68^ it was found that some e‐cigarettes sold in Nigeria had misleading or insufficient information on their nicotine content. Aside from this study,^68^ there is no other study that has investigated issues related to e‐cigarette regulations in Nigeria; hence, there is a need to expand on the existing literature in this area. Notably, some studies outside Nigeria have examined regulations of e‐cigarettes^51^, ^52^, ^53^ including the geospatial spread of vape shops and its relation to e‐cigarette use, especially among adolescents and young adults.^77^, ^78^ Considering the huge relevance of these studies to policy formulation and implementation, it is highly recommended that similar studies be conducted in Nigeria. This area of future research should be considered a priority as e‐cigarette regulation and laws may predict the patterns of e‐cigarette use, its availability, advertising, marketing, and distribution to potential end‐users. A key area of public health significance is e‐cigarette advertising, marketing, and sponsorship. Several studies outside Nigeria have reported the impact of e‐cigarette advertising and marketing on attempts to quit vaping^48^, ^49^, ^50^ and the potential to encourage individuals who have quit smoking to start vaping.^79^, ^80^ Hence, more studies are needed to provide scientific evidence on e‐cigarette advertising, marketing, and sponsorship in Nigeria, as such evidence is pivotal for proper planning, implementation, and evaluation of policies on e‐cigarettes in Nigeria.

## 5 CONCLUSION

This scoping review has described available information on vaping practices in Nigeria while highlighting several limitations on e‐cigarettes and vaping research in Nigeria especially as it relates to its knowledge and awareness, prevalence, determinants, health implications, program or policy interventions, and regulations. This review has identified determinants of vaping in Nigeria which include being a youth, having a health condition, severe anxiety, tobacco use, peer influence, and current alcohol use. There is limited information to highlight the trends of e‐cigarette prevalence in Nigeria. There is no evidence of a national strategy or regulations to tackle rising e‐cigarette use in Nigeria nor are there available evidence‐based programs to address or limit its use. This scoping review highlights the need for more evidence on the knowledge, awareness, and prevalence of vaping in Nigeria. There is a need for future research to better understand the complex interplay of determinants that affect vaping, the need to advance specific evidence of the health implications of vaping, and evidence‐based clinical and public health strategies to limit them. There should be more coordinated and virile approaches towards e‐cigarette regulation, and other policies that may impact vaping, encourage vaping cessation, prevent addiction, and reduce harm, especially among the at‐risk e‐cigarette users in Nigeria.

## AUTHOR CONTRIBUTIONS

**Oluwatobi E. Adegbile**: Conceptualization; data curation; formal analysis; investigation; methodology; project administration; resources; software; validation; visualization; writing—original draft; writing—review and editing. **Oluwatomilayo D. Adeniji**: Project administration; resources; writing—review and editing. **Jimoh Amzat**: Resources; writing—original draft; writing—review and editing. **Kehinde K. Kanmodi**: Conceptualization; data curation; formal analysis; funding acquisition; investigation; methodology; project administration; resources; software; supervision; validation; visualization; writing—original draft; writing—review and editing.

## CONFLICT OF INTEREST STATEMENT

Kehinde K. Kanmodi is an Editorial Board member of *Health Science Reports* and a coauthor of this article. To minimize bias, they were excluded from all editorial decision‐making related to the acceptance of this article for publication. The remaining authors declare no conflict of interest.

## TRANSPARENCY STATEMENT

The lead author Kehinde K. Kanmodi affirms that this manuscript is an honest, accurate, and transparent account of the study being reported; that no important aspects of the study have been omitted; and that any discrepancies from the study as planned (and, if relevant, registered) have been explained.

## Supporting information

Supporting information.

## Data Availability

Data sharing does not apply to this article as no new data were created or analyzed in this study.
