# Supplementary material for: E‐cigarettes in Nigeria: A scoping review of evidence
Source: Health Sci Rep. 2024 Apr 24;7(4):e2074. doi: 10.1002/hsr2.2074 (PMC11040567; doi:10.1002/hsr2.2074)
Supplement: Supplementary file 1 — Supporting information. [file HSR2-7-e2074-s001.docx]

**SUPPLEMENTARY FILE**

**Table S1. Search string for PubMed database search**

| **Tag** | **Subject search** | **Search String** |
| --- | --- | --- |
| #1 | E-cigarette | (((E-cigarette[Title/Abstract]) OR (Vape[Title/Abstract])) OR (Vaping[Title/Abstract])) OR (electronic nicotine delivery[Title/Abstract]) |
| #2 | Nigeria | Nigeria[Title/Abstract] |
| #3 | #1 AND #2 | (#1) AND (#2) |

**Table S2. Search string for SCOPUS database search**

| **Tag** | **Subject search** | **Search String** |
| --- | --- | --- |
| #1 | E-cigarette | ( TITLE-ABS-KEY ( e-cigarette )  OR  TITLE-ABS-KEY ( vape )  OR  TITLE-ABS-KEY ( vaping )  OR  TITLE-ABS-KEY ( electronic  AND nicotine  AND delivery ) ) |
| #2 | Nigeria | TITLE-ABS-KEY ( Nigeria ) |
| #3 | #1 AND #2 | (#1) AND (#2) |

**Table S3. Search string for other databases (CINAHL Complete and APA PsycInfo) search via EBSCO interface**

| **Tag** | **Subject search** | **Search String** |
| --- | --- | --- |
| S1 | E-cigarette | AB E-cigarette OR AB vape OR AB vaping OR AB electronic nicotine delivery |
| S2 | Nigeria | AB Nigeria |
| S3 | S1 AND S2 | (S1) AND (S2) |

**Table S4. List of publications whose full text were screened and their decision outcomes**

| **S/N** | **Citation** | **Screening Outcome** | |
| --- | --- | --- | --- |
|  |  | Include | Exclude (with reasons) |
| 1 | Erinoso O, Oyapero A, Amure M, Osoba M, Osibogun O, Wright K, Osibogun A. Electronic cigarette use among adolescents and young adults in Nigeria: Prevalence, associated factors and patterns of use. PLoS One. 2021 Oct 22;16(10):e0258850. doi: 10.1371/journal.pone.0258850. PMID: 34679087; PMCID: PMC8535460. | Yes |  |
| 2 | Erinoso OA, Osibogun O, Egbe CO, Wright O, Oyapero A, Osibogun A. Electronic nicotine delivery systems in Nigeria: product types, flavours and nicotine content labels. Tob Control. 2022 Aug 17:tobaccocontrol-2022-057578. doi: 10.1136/tc-2022-057578. Epub ahead of print. PMID: 35977822. | Yes |  |
| 3 | Alade O, Folayan MO, Adeniyi A, Adeyemo YI, Oyapero A, Olatosi OO, Nzomiwu C, Popoola BO, Eigbobo J, Oziegbe E, Oyedele T, El Tantawi M, Sabbagh HJ. Differences in Oral Lesions Associated with Tobacco Smoking, E-Cigarette Use and COVID-19 Infection among Adolescents and Young People in Nigeria. Int J Environ Res Public Health. 2022 Aug 24;19(17):10509. doi: 10.3390/ijerph191710509. PMID: 36078225; PMCID: PMC9517769. | Yes |  |
| 4 | Omaiye EE, Cordova I, Davis B, Talbot P. Counterfeit Electronic Cigarette Products with Mislabeled Nicotine Concentrations. Tob Regul Sci. 2017 Jul;3(3):347-357. doi: 10.18001/TRS.3.3.10. Epub 2017 Jul 1. PMID: 29744375; PMCID: PMC5937541. | Yes |  |
| 5 | Osibogun O, Odukoya OO, Odusolu YO, Osibogun A. Knowledge and risk perception of e-cigarettes and hookah amongst youths in Lagos State, Nigeria: An exploratory study. Niger Postgrad Med J. 2020 Oct-Dec;27(4):384-390. doi: 10.4103/npmj.npmj_261_20. PMID: 33154294. | Yes |  |
| 6 | Folayan MO, Alade O, Adeyemo Y, Sabbagh HJ, Oyapero A, Oziegbe EO, Popoola BO, Quritum M, El Tantawi M. Differences in risk indicators associated with electronic cigarette use and tobacco smoking among adolescents and young people in Nigeria. BMJ Open Respir Res. 2022 Sep;9(1):e001285. doi: 10.1136/bmjresp-2022-001285. PMID: 36109086; PMCID: PMC9478830. | Yes |  |

**Table S5. Quality appraisal outcomes of the appraised quantitative non-randomized studies using the Mixed Methods Appraisal Tool**

| **No.** | **Author (Year)** | **Study Design** | **Responses to the Appraisal Questions for Quantitative Non-randomized Studies** | | | | | | | **Scored Points (out of a Total of 7 Points)** | **Grade** |
| --- | --- | --- | --- | --- | --- | --- | --- | --- | --- | --- | --- |
|  |  |  | Are there clear research questions? | Do the collected data allow to address the research questions? | Are the participants representative of the target population? | Are measurements appropriate regarding both the outcome and intervention (or exposure)? | Are there complete outcome data? | Are the confounders accounted for in the design and analysis? | During the study period, is the intervention administered (or exposure occurred) as intended? |  |  |
| 1 | Alade et al. (2022) | Cross-sectional analytical study | Yes | Yes | I can’t tell | Yes | Yes | Yes | Yes | 6/7 | Above average |
| 2 | Folayan et al. (2022) | Cross-sectional analytical study | Yes | Yes | I can’t tell | Yes | Yes | Yes | Yes | 6/7 | Above average |
| 3 | Erinoso et al. (2021) | Cross-sectional analytical study | Yes | Yes | I can’t tell | Yes | Yes | Yes | Yes | 6/7 | Above average |
| Yes – 1 point; No – 0 point; I can’t tell – 0 point; Above average – 4/7 points and above; Below average – 3/7 points and below | | | | | | | | | | | |

**Table S6. Quality appraisal outcomes of the appraised quantitative descriptive study using the Mixed Methods Appraisal Tool**

| **No.** | **Author (Year)** | **Study Design** | **Responses to the Appraisal Questions for Quantitative Descriptive Studies** | | | | | | | **Scored Points (out of a Total of 7 Points)** | **Grade** |
| --- | --- | --- | --- | --- | --- | --- | --- | --- | --- | --- | --- |
|  |  |  | Are there clear research questions? | Do the collected data allow to address the research questions? | Is the sampling strategy relevant to address the research question? | Is the sample representative of the target population? | Are the measurements appropriate? | Is the risk of nonresponse bias low? | Is the statistical analysis appropriate to answer the research question? |  |  |
| 1 | Omaiye et al (2017) | Cross-sectional descriptive study | Yes | Yes | Yes | I can’t tell | Yes | Yes | Yes | 6/7 | Above average |
| 2 | Erinoso et al (2022) | Cross-sectional descriptive study | Yes | Yes | Yes | I can’t tell | Yes | Yes | Yes | 6/7 | Above average |
| Yes – 1 point; No – 0 point; I can’t tell – 0 point; Above average – 4/7 points and above; Below average – 3/7 points and below | | | | | | | | | | | |

**Table S7. Quality appraisal outcomes of the appraised qualitative study using the Mixed Methods Appraisal Tool**

| **No.** | **Author (Year)** | **Study Design** | **Responses to the Appraisal Questions for Qualitative Studies** | | | | | | | **Scored Points (out of a Total of 7 Points)** | **Grade** |
| --- | --- | --- | --- | --- | --- | --- | --- | --- | --- | --- | --- |
|  |  |  | Are there clear research questions? | Do the collected data allow to address the research questions? | Is the qualitative approach appropriate to answer the research question? | Are the qualitative data collection methods adequate to address the research question? | Are the findings adequately derived from the data? | Is the interpretation of results sufficiently substantiated by data? | Is there coherence between qualitative data sources, collection, analysis and interpretation? |  |  |
| 1 | Osibogun et al. (2020) | Qualitative study | Yes | Yes | Yes | Yes | Yes | Yes | Yes | 7/7 | Above average |
| Yes – 1 point; No – 0 point; I can’t tell – 0 point; Above average – 4/7 points and above; Below average – 3/7 points and below | | | | | | | | | | | |
